# A convolutional neural network for steady state visual evoked potential classification under ambulatory environment

**DOI:** 10.1371/journal.pone.0172578

**Published:** 2017-02-22

**Authors:** No-Sang Kwak, Klaus-Robert Müller, Seong-Whan Lee

**Affiliations:** 1 Department of Brain and Cognitive Engineering, Korea University, Anam-dong, Seongbuk-ku, Seoul, Republic of Korea; 2 Department of Computer Science, TU Berlin, Berlin, Germany; Ulm University, GERMANY

## Abstract

The robust analysis of neural signals is a challenging problem. Here, we contribute a convolutional neural network (CNN) for the robust classification of a steady-state visual evoked potentials (SSVEPs) paradigm. We measure electroencephalogram (EEG)-based SSVEPs for a brain-controlled exoskeleton under ambulatory conditions in which numerous artifacts may deteriorate decoding. The proposed CNN is shown to achieve reliable performance under these challenging conditions. To validate the proposed method, we have acquired an SSVEP dataset under two conditions: 1) a static environment, in a standing position while fixated into a lower-limb exoskeleton and 2) an ambulatory environment, walking along a test course wearing the exoskeleton (here, artifacts are most challenging). The proposed CNN is compared to a standard neural network and other state-of-the-art methods for SSVEP decoding (i.e., a canonical correlation analysis (CCA)-based classifier, a multivariate synchronization index (MSI), a CCA combined with k-nearest neighbors (CCA-KNN) classifier) in an offline analysis. We found highly encouraging SSVEP decoding results for the CNN architecture, surpassing those of other methods with classification rates of 99.28% and 94.03% in the static and ambulatory conditions, respectively. A subsequent analysis inspects the representation found by the CNN at each layer and can thus contribute to a better understanding of the CNN’s robust, accurate decoding abilities.

## Introduction

A brain-computer interface (BCI) allows for the decoding of (a limited set of) user intentions employing only brain signals—without making use of peripheral nerve activity or muscles [1, 2]. BCIs allow users to harness brain states for driving devices such as spelling interfaces [3–5], wheelchairs [6, 7], computer games [8, 9] or other assistive devices [10–12]. Recent BCI studies have demonstrated the possibility of decoding the user’s intentions within a virtual reality environment [13] and using an exoskeleton [14–17]. Others have investigated the decoding of expressive human movement from brain signals [18]. Furthermore, researchers have developed several applications of BCI systems for the rehabilitation of stroke patients [19–23].

Existing EEG-based BCI techniques generally use EEG paradigms such as the modulation of sensorimotor rhythms through motor imagery (MI) [24–26]; event related potentials (ERPs), including P300 [27, 28]; and steady-state visual-, auditory-, or somatosensory-evoked potentials (SSVEPs [29, 30], SSAEPs [31, 32], and SSSEPs [33, 34]).

Of these EEG paradigms, SSVEPs have shown reliable performance in terms of accuracy and response time, even with a small number of EEG channels, at a relatively high information transfer rate (ITR) [35] and reasonable signal-to-noise ratio (SNR) [36]. SSVEPs are periodic responses elicited by the repetitive fast presentation of visual stimuli; they typically operate at frequencies between 1 and 100 Hz and can be distinguished by their characteristic composition of harmonic frequencies [33, 37].

Various machine learning methods are used to detect SSVEPs: first and foremost, classifiers based on canonical correlation analysis (CCA), a multivariate statistical method for exploring the relationships between two sets of variables, can harvest the harmonic frequency composition of SSVEPs. CCA detects SSVEPs by finding the weight vectors that maximize the correlations between the two datasets. In our SSVEP paradigm, the maximum correlation extracted by CCA is used to detect the respective frequencies of the visual stimuli to which the subject attended [37]. Modified CCA-based classifiers have been introduced, such as a multiway extension of CCA [38], phase-constrained [39] and multiset [40] CCA methods. In addition, stimulus-locked intertrace correlation (SLIC) [41] and the sparsity-inducing LASSO-based method [42] have been proposed for SSVEP classification. The multivariate synchronization index (MSI) was introduced to estimate the synchronization between two signals as an index for decoding stimulus frequency [43, 44]. SSVEP decoding can be further extended by employing characteristics based on phase and harmonics [35], boosting the ITRs significantly. Recently, deep-learning-based SSVEP classification methods [45–47] have also been considered; however, all have thus far used prestructuring by employing a Fourier transform in the CNN layer.

Recently, brain machine interface (BMI) researchers have turned their focus to connecting SSVEP-based BMIs to mobile systems with wireless EEG telemetry [14, 48] in order to explore the feasibility of implementing online SSVEP-based BMIs. This progress has greatly facilitated the transition of laboratory-oriented BMI systems to more practical ambulatory brain-controlled exoskeletons [14].

Despite the technical and machine learning progress outlined, systematic performance deterioration between ambulatory and static BMI-control conditions has been found [49, 50], primarily because of the artifacts, which are caused by subject’s motion, head swing, walking speed or sound, and the exoskeleton’s electric motors [51, 52]; these artifacts may, in addition, differ across users [14].

We address this key challenge by exploring deep learning methods as a means to reliably minimize the influence of artifacts on ambulatory SSVEP-BMIs. Here, we consider as artifacts all signals that have non-cerebral origin and that might mimic non-task-related cognitive signals or that are induced by external factors (e.g., while walking, a subject’s head may be moved by the exoskeleton which can give rise to swinging movements in the line between the electrodes and EEG amplifiers, leading to disconnections or high impedance measurements in extreme cases). These artifacts typically distort the analysis of an EEG.

Therefore, we propose a CNN-based classifier that uses frequency features as input for robust SSVEP detection in ambulatory conditions. In the course of the CNN training process, the model learns an appropriate representation for solving the problem [53, 54]. The receptive field/convolution kernel structure of the trained model can then be analyzed, and we can interpret the high-level features found by the deep network as we inspect each layer. Our CNN architecture compares favorably with standard neural network and other state-of-the-art methods used in ambulatory SSVEP BMIs.

## Materials and methods

### Experiment

#### Experimental environment

**Fig 1:**
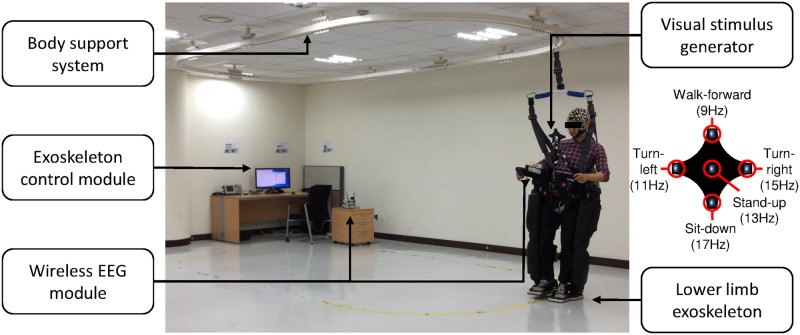
Experimental environment. Subject wearing the lower-limb exoskeleton and focusing on an LED from the visual stimulus generator. The EEG is transferred by a wireless interface to the PC. A body support system (a rail beneath the ceiling) was connected to the subject for safety purposes. The exoskeleton was controlled by an external operator using a keyboard controller.

We designed an experimental environment for SSVEP-based exoskeleton control following [14]. In particular, we used a powered lower-limb exoskeleton (Rex, Rex Bionics Ltd.) with a visual stimulus generator attached to the robot for stimulating SSVEPs in an ambulatory environment. The visual stimulus generator presented visual stimuli using five light-emitting diodes (LEDs: 9, 11, 13, 15, and 17 Hz with a 0.5 duty ratio) which were controlled by a micro controller unit (MCU; Atmega128), as shown in Fig 1.

**Fig 2:**
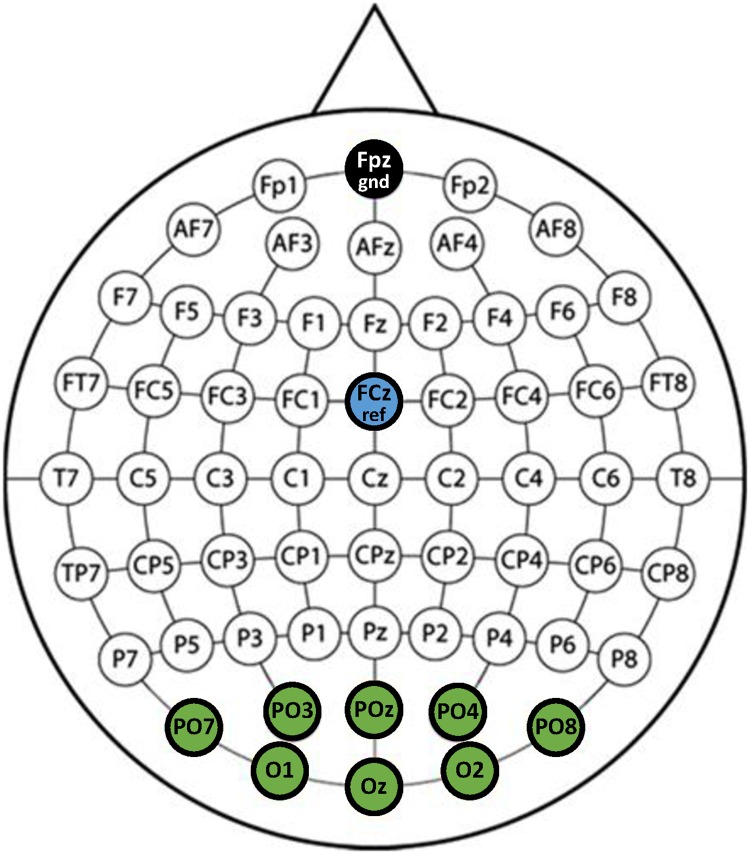
EEG channel layout. Channel layout using 8 channels (PO7, PO3, PO, PO4, PO8, O1, Oz, and O2) for SSVEP acquisition with a reference (FCz) and ground (Fpz).

The EEG was acquired from a wireless interface (MOVE system, Brain Products GmbH) using 8 Ag/AgCl electrodes at locations PO7, PO3, PO, PO4, PO8, O1, Oz, and O2, with reference (FCz) and ground (Fpz) electordes, illustrated in Fig 2. Impedances were maintained below 10 kΩ and the sampling frequency rate was 1 kHz. A 60 Hz notch filter was applied to the EEG data for removing AC power supply noise.

#### Subject

Seven healthy subjects, with normal or corrected-to-normal vision and no history of neurological disease, participated in this study (age range: 24–30 years; 5 males, 2 females). The experiments were conducted in accordance with the principles expressed in the Declaration of Helsinki. This study was reviewed and approved by the Institutional Review Board at Korea University [1040548-KU-IRB-14–166-A-2] and written informed consent was obtained from all participants before the experiments.

#### Experiment tasks

**Fig 3:**
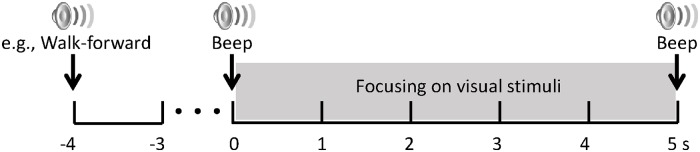
Experimental procedure for Task 1 and 2. After a random auditory cue, a start sound follows 3 s later; then, the subjects attended the corresponding LED for 5 s. All LEDs blinked at differing frequencies during the tasks.

We acquired two SSVEP datasets under static and ambulatory conditions, respectively, to compare the performance of the SSVEP classifiers. From Task 1, we collected SSVEP data with the exoskeleton in a standing position (static SSVEP). In Task 2 (ambulatory SSVEP), the SSVEP signals were acquired while the exoskeleton was walking. In both tasks, we performed the experimental procedure described in Fig 3. After the random auditory cue was given, a start sound was presented 3 s later, and then the subjects attended the corresponding visual stimuli for 5 s. The auditory cue was given in random order to prevent potentially biased results for the stimulation frequency, the start sound gave the subjects time to prepare to focus on the visual stimuli. The auditory cues were guided by voice recordings to indicate commands such as “walk forward”, “turn left”, “turn right”, “sit”, and “stand”, and were approximately 1 s in length. Note that during the experimental tasks, all LEDs were blinking simultaneously at different frequencies.

- Task 1 (Static SSVEP): The subjects were asked to focus their attention on the visual stimulus in a standing position while wearing the exoskeleton. Corresponding visual stimuli were given by auditory cue and 50 auditory cues were presented in total (10 times in each class).
- Task 2 (Ambulatory SSVEP): The subjects were asked to focus on visual stimuli while engaged in continuous walking using the exoskeleton. The exoskeleton was operated by a wireless controller, per the decoded intention of the subject. a total of 250 auditory cues were presented (50 in each class).

### Neural network architectures

We now investigate three neural network architectures for SSVEP decoding, CNN-1 and CNN-2, which use convolutional kernels, and NN, standard feedforward neural network without convolution layers.

We show that CNN-1 has the best classification rate; in CNN-2, we included a fully connected layer with 3 units for visualizing feature representations as a function of the learning progress.

#### Input data

The acquired EEG data were preprocessed for CNN learning by band-pass filtering from 4–40 Hz. Then, the filtered data were segmented using a 2 s sliding window (2,000 time samples × 8 channels). The segmented data were transformed using a fast Fourier transform (FFT). Then, we used 120 samples from each channel, corresponding to 5–35 Hz. Finally, data were normalized to the range from 0 to 1. Therefore, the input data dimension for CNN learning was 120 frequency samples (*N*_*fs*_) by 8 channels (*N*_*ch*_). The number of input data for training depends on the experimental task and is therefore described in the Evaluation section.

#### Network architecture overview

**Fig 4:**
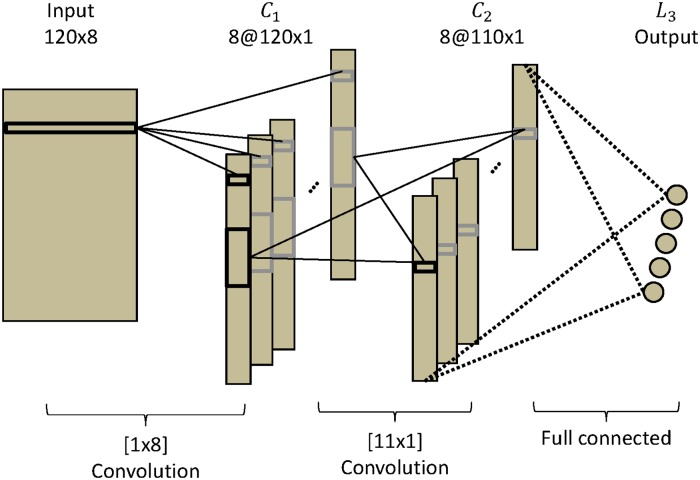
CNN-1 architecture. CNN-1 is composed of three layers, two convolutional layers and an output layer.

The **CNN-1** network has three layers, each composed of one or several maps that contain frequency information for the different channels (similar to [55]). The input layer is defined as *I*_*p*, *j*_ with 1 ≤ *p* ≤ *N*_*fs*_ and 1 ≤ *j* ≤ *N*_*ch*_; here, *N*_*fs*_ = 120 is the number of frequency samples and *N*_*ch*_ = 8 is the number of channels. The first and second hidden layers are composed of *N*_*ch*_ maps. Each map in *C*_1_ has size *N*_*fs*_; each map in *C*_2_ is composed of 110 units. The output layer has 5 units, which represent the five classes of the SSVEP signals. This layer is fully connected to *C*_2_ as in Fig 4.

**Fig 5:**
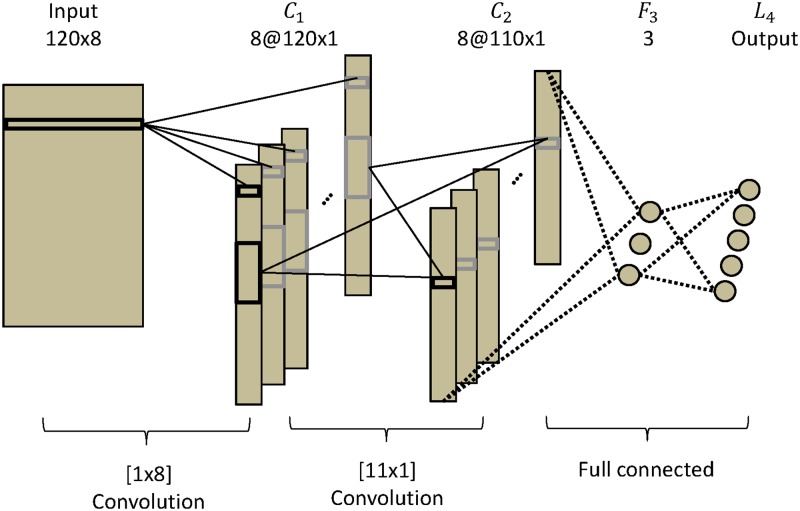
CNN-2 architecture. CNN-2 is composed of four layers: two convolutional layers, one fully connected layer, and an output layer.

The **CNN-2** network is composed of four layers. The input layer is defined as *I*_*p*, *j*_ with 1 ≤ *p* ≤ *N*_*fs*_ and 1 ≤ *j* ≤ *N*_*ch*_. The first and second hidden layers are composed of *N*_*ch*_ maps. Each map in *C*_1_ has size *N*_*fs*_. Each map of *C*_2_ has 110 units. To this point, CNN-2 is equivalent to CNN-1. The difference comes in the third hidden layer *F*_3_, which is fully connected and consist of 3 units. The each unit is fully connected to *C*_2_. The output layer has 5 units that represent the five classes of SSVEP. This layer is fully connected to *F*_3_. The 3 units in *F*_3_ are used to visualize the properties of the representation that CNN-2 has learned, as depicted in Fig 5.

**Fig 6:**
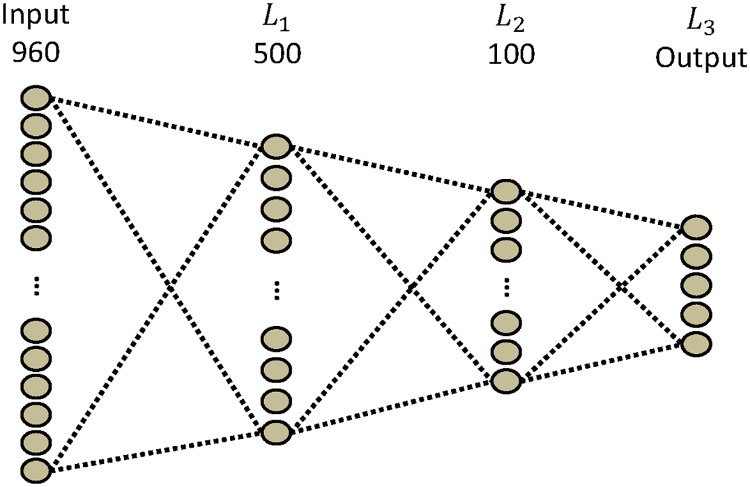
NN architecture. The NN is composed of three layers: two fully connected layers and an output layer.

The standard **NN** is composed of three layers. For the input layer, we concatenated the 120 by 8 input into a 960-unit vector. The first hidden layer is composed of 500 units, the second has 100 units, and the output layer has 5 units to represent the five classes. All layers are fully connected, as in Fig 6.

#### Learning

A unit in the network is defined by $x_{k}^{l}\left(p\right)$, where *l* is the layer, *k* is the map, and *p* is the position of the unit in the map,

$$\begin{array}{c} x_{k}^{l}\left(p\right)=f\left(\sigma_{k}^{l}\left(p\right)\right), \end{array}$$ (1)

where *f* is the classical sigmoid function used for the layers:

$$\begin{array}{c} f\left(\sigma \right)=\frac{1}{1+exp^{-\sigma }}. \end{array}$$ (2)

$\sigma_{k}^{l}\left(p\right)$ represents the scalar product of a set of input units and the weight connections between these units and the unit number of *p* in map *k* in layer *l*. For *C*_1_ and *C*_2_, which are convolutional layers, each unit of the map shares the same set of weights. The units of these layers are connected to a subset of units fed by the convolutional kernel from the previous layer. Instead of learning one set of weights for each unit, where the weights depend on unit position, the weights are learned independently to their corresponding output unit. *L*_3_ is the output layer in CNN-1 and *L*_4_ is the output layer in CNN-2.

- CNN-1
  — For *C*_1_:
    $$\begin{array}{c} \sigma_{k}^{1}\left(p\right)=w\left(1,k,0\right)+\sum_{j=1}^{j\leq N_{ch}}I_{p,j}w\left(1,k,j\right), \end{array}$$ (3)
    where *w*(1, k, 0) is a bias and *w*(1, k, j) is a set of weights with 1 ≤ *j* ≤ *N*_*ch*_. In this layer, there are *N*_*ch*_ weights for each map. The convolution kernel has a size of 1 × *N*_*ch*_.
  — For *C*_2_:
    $$\begin{array}{c} \sigma_{k}^{2}\left(p\right)=w\left(2,k,0\right)+\sum_{i=1}^{i\leq N_{ch}}\sum_{j=1}^{j\leq 11}x_{i}^{1}\left(p+j-1\right)w\left(2,i,j\right), \end{array}$$ (4)
    where *w*(2, k, 0) is a bias. This layer transforms the signal of 120 units into 110 new values in *C*_2_, reducing the size of the signal to analyze while applying an identical linear transformation to the 110 units of each map. This layer translates spectral filters. The convolution kernel has a size of 11 × 1.
  — For *L*_3_:
    $$\begin{array}{c} \sigma^{3}\left(p\right)=w\left(3,0,p\right)+\sum_{k=1}^{k\leq 8}\sum_{l=1}^{l\leq 110}x_{k}^{2}\left(l\right)w\left(3,k,l\right), \end{array}$$ (5)
    where *w*(3, 0, p) is a bias. Each unit of *L*_3_ is connected to each unit of *C*_2_.
- CNN-2
  — *C*_1_ and *C*_2_ are the same as in CNN-1.
  — For *F*_3_:
    $$\begin{array}{c} \sigma^{3}\left(p\right)=w\left(3,0,p\right)+\sum_{k=1}^{k\leq 8}\sum_{l=1}^{l\leq 110}x_{k}^{2}\left(l\right)w\left(3,k,l\right), \end{array}$$ (6)
    where *w*(3, 0, p) is a bias. Each unit of *F*_3_ is connected to each unit of *C*_2_
  — For *L*_4_:
    $$\begin{array}{c} \sigma^{4}\left(p\right)=w\left(4,0,p\right)+\sum_{l=1}^{l\leq 3}x^{3}\left(l\right)w\left(4,l\right), \end{array}$$ (7)
    where *w*(4, 0, p) is a bias. Each unit of *L*_4_ is connected to each unit of *F*_3_

The gradient descent learning algorithm uses standard error backpropagation to correct the network weights [56–58]. The learning rate was 0.1 and weights were initialized with a normal distribution on the interval [-sqrt(6/(*N*_*in*_+*N*_*out*_)), sqrt(6/(*N*_*in*_+*N*_*out*_))], where *N*_*in*_ is the number of input weights and *N*_*out*_ is the number of output weights following [58]. The number of learning iterations was 50, but training stopped once the decrease of in error rate was smaller than 0.5% after 10 iterations.

### Evaluation

In this section, we validate the three neural networks (CNN-1, CNN-2, and NN), and compare them to previously used methods: CCA [37, 48], MSI [43] and CCA combined with *k*-nearest neighbors (CCA-KNN) [14].

For each classifier, we compute the 10-fold cross-validation error, splitting the data chronologically (a common method in EEG classification) to preserve the data’s non-stationarity and avoid overfitting [27, 59]. For the test data, both datasets (50 trials of 5 s for static SSVEPs and 250 trials for ambulatory SSVEPs, randomly permuted) were segmented using a 2 s sliding window with a 10 ms shift size, segmenting a 5 s trial into three hundred 2 s trials. As a result, there were 1,500 static and 7,500 ambulatory SSVEP test data points in each fold. Deep neural networks generally show higher performance for larger amounts of data [53]. Hence, we tested the classifiers with different training data sizes; in particular, different segmentations of the data were considered. Using a 2 s sliding window with different shift sizes (60, 30, 20, 15, 12, and 10 ms), we obtained a trial segmentation into 50, 100, 150, 200, 250, and 300 data samples. Thus, there were 2,250, 4,500, 6,750, 9,000, 11,250, and 13,500 training data for the static SSVEPs, and 11,250, 22,500, 33,750, 45,000, 56,250, and 67,500 for the ambulatory SSVEPs. Note that although we used a small size shift, there was no overlap between training and test data in order to prevent overfitting. The CCA method does not require a training phase. Thus, we only show its results on test data.

We now briefly describe the CCA, CCA-KNN, and MSI methods. CCA is a multivariate statistical method [60, 61] that finds a pair of linear combinations such that the correlation between two canonical variables *X* and *Y* is maximized. As *X*(*t*), we chose 2 s EEG windows; as *Y*_*i*_(*t*), we use the five reference frequencies (*f*_1_ = 9, *f*_2_ = 11, …,*f*_5_ = 17) from the five visual stimuli [14]

$$\begin{array}{c} Y_{i}\left(t\right)={\left(sin(2\pi f_{i}t),cos(2\pi f_{i}t),sin(2\pi \left(2f_{i}\right)t),cos(2\pi \left(2f_{i}\right)t)\right)}^{\prime },\;t=\frac{1}{S},\frac{2}{S},\cdots ,\frac{T}{S}, \end{array}$$ (8)

where *T* is the number of sampling points and *S* denotes the sampling rate. CCA finds weight vectors, *W*_*x*_ and *W*_*y*_, that maximize the correlation between the canonical variants *x* = *X*′*W*_*x*_ and *y* = *Y*′*W*_*y*_, by solving

$$\begin{array}{c} \max_{W_{x}W_{y}}\rho \left(x,y\right)=\frac{E[x^{\prime }y]}{\sqrt{E\left[x^{\prime }x\right]E\left[y^{\prime }y\right]}}=\frac{E[W_{x}^{\prime }XY^{\prime }W_{y}]}{\sqrt{E\left[W_{x}^{\prime }XX^{\prime }W_{x}\right]E\left[W_{Y}^{\prime }YY^{\prime }W_{y}\right]}}. \end{array}$$ (9)

The maximum *ρ* with respect to *W*_*x*_ and *W*_*y*_ is the maximum canonical correlation. The canonical correlation *ρ*_*f*_*i*__, where i = 1, …, 5, is used for detecting the frequency of the LED that a subject is attending by

$$\begin{array}{c} O_{i}=\max_{i}\left(\rho_{f_{i}}\right),\;i=1,\ldots ,5, \end{array}$$ (10)

where *O*_*i*_ are the output classes corresponding to the five visual stimuli.

For CCA-KNN [14], the set of canonical correlations (***ρ*** = (*ρ*_*f*_1__, …, *ρ*_*f*_5__)) is used as a feature vector for subsequent KNN classification, each with a class label. In the training step, the algorithm consists only of storing the feature vectors and class labels of the training samples. In classification, an unlabeled vector is classified by assigning the label to the most frequent of the *k* nearest training samples, where the Euclidean distance is used as a distance metric.

For MSI [43, 44], the *S*-estimator, based on the entropy of the normalized eigenvalues of the correlation matrix of multivariate signals, was used as the index. Thus, MSI creates a reference signal from the stimulus frequencies used in an SSVEP-based BCI system similarly to CCA.

## Results and discussion

EEG signals are highly variable across subjects and experimental environments (see Figs 5 and 6 and Tables 1 and 2 in [14]). The SSVEP signals acquired from the static exoskeleton show more pronounced frequency information than in the ambulatory environment. In the static SSVEP, we can observe the increased frequency components that are visible at the stimulus frequency. In the ambulatory SSVEP, however, because of the higher artifactual content, this effect becomes less clearly visible (see S1 Fig for selected input and average data under both conditions).

### Static SSVEP

In Table 1, we show the 10-fold cross-validation results for 13,500 training data validated on 1,500 test data points for all subjects. CNN-1 showed the best classification accuracy of all subjects in each classifier. For low-performing subjects, with a CCA accuracy under 80% in the ambulatory SSVEP (i.e., subjects S3–7, see Table 3), the neural network results stayed robust. Clearly, the CCA method exhibits significantly lower performance.

**Table 1:** 10-fold cross validation results of individual subjects with the maximum quantity of training data (i.e., 13,500) using static SSVEP.

	S1	S2	S3	S4	S5	S6	S7	Low	All
CCA	91.27 (-8.59)	93.78 (-6.84)	76.14 (-22.72)	92.86 (-6.3)	67.85 (-31.62)	78.22 (-20.39)	82.56 (-16.77)	79.53±9.17 (-19.55)	83.24±9.84 (-16.04)
MSI	91.54 (-8.32)	95.71 (-3.99)	78.74 (-20.12)	94.67 (-4.49)	70.61 (-28.85)	79.34 (-19.27)	83.43 (-15.91)	81.86±8.77 (-17.73)	84.86±9.41 (-14.42)
CCA-KNN	100±0 (+0.14)	99.80±0.27 (+0.1)	93.91±2.11 (-4.95)	98.97±1.11 (-0.19)	99.13±0.95 (-0.34)	93.26±3.70 (-5.35)	98.86±0.91 (-0.47)	96.83±2.97 (-2.25)	97.70±2.85 (-1.58)
NN	98.95±1.51 (-1.01)	98.85±1.9 (-0.85)	97.53±2.95 (-1.33)	98.87±1.28 (-0.29)	99.73±0.70 (+0.26)	97.11±3.42 (-1.5)	98.01±2.68 (-1.32)	98.25±1.05 (-0.64)	98.44±0.92 (-0.84)
CNN-2	98.46±1.97 (-1.4)	98.17±2.16 (-1.53)	97.90±2.24 (-0.96)	97.43±2.15 (-1.73)	99.51±0.70 (+0.04)	96.54±3.18 (-2.07)	97.50±2.75 (-1.83)	97.63±1.54 (-1.45)	97.83±1.31 (-1.45)
CNN-1	99.86±0.27	99.70±0.27	98.86±1.33	99.16±1.77	99.47±0.76	98.61±2.30	99.33±0.72	99.08±0.35	99.28±0.45

10-fold cross validation results of static SSVEP classification for 13,500 training data points with 1,500 test data for all subjects. Low indicates subjects who have a low CCA accuracy (under 80% in the ambulatory SSVEP, i.e., subjects S3–7). Parentheses indicate accuracy the difference compared with CNN-1.

**Fig 7:**
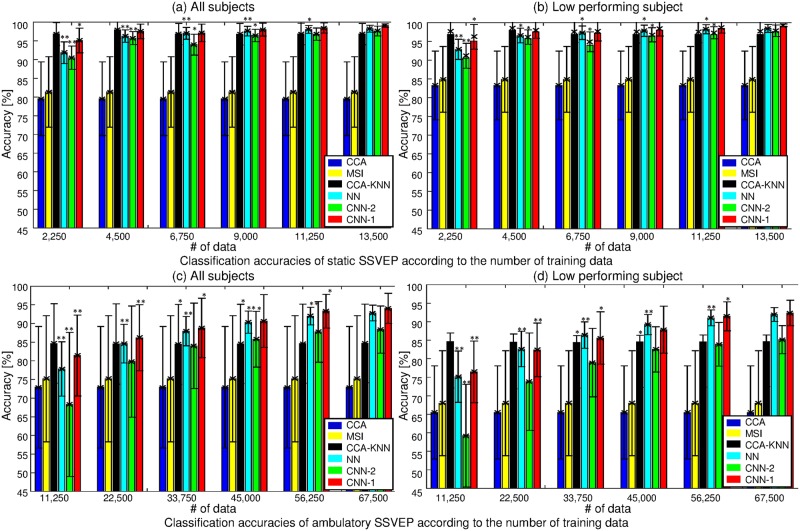
10-fold cross-validation performance comparison using static (top) and ambulatory SSVEP (bottom) as the number of training data increases. (a) Average of all subjects in static SSVEP. (b) Low performing subjects. 1,500 test data were used for each fold. One asterisk indicates indicates the 5% significance level between corresponding to the number of training data samples and 13,500 training data samples. Two asterisks are the 1% significance level. (c) Average of all subjects in ambulatory SSVEP. (d) Low performing subjects. 7,500 test data were used for each fold. One asterisk indicates indicates the 5% significance level between the corresponding number of training data samples and 67,500 training data samples. Two asterisks are the 1% significance level.

With fewer training data (see Table 2 and Fig 7 (top) for the 10-fold cross-validation results of the static task), we observe a decaying performance for the neural networks, which is to be expected. Note that the CCA and MSI methods stay essentially constant as a function of data, since no training phase is required because the canonical correlations and synchronization index with reference signals are simply computed in order to find the maximum value. The CCA-KNN classifier was trained for *k* = 1, 3, 5, and 7, respectively, and *k* was selected on the training set to achieve the best accuracy. Fig 7 (top) presents the average accuracy of each classifier as a function of the number of training data for all subjects (a) and low-performing subjects (b). Statistical analysis of these results shows a significant improvement with larger training data sizes for the neural network classifiers. We provide more information on the difference between CNN-1 and the other methods for all subjects and low-performing subjects in S2(a) and S2(b) Fig, respectively. CNN-1 outperforms other classifiers; however, CCA-KNN shows better classification results for 4,500 training data samples or fewer, as we can see from the positive values in brackets.

**Table 2:** 10-fold cross-validation of static SSVEP classification by the quantity of training data.

	2,250		4,500		6,750		9,000		11,250		13,500	
	Low	All	Low	All	Low	All	Low	All	Low	All	Low	All
CCA-KNN	96.85 (+1.76)	97.71 (+1.51)	97.91 (+0.38)	98.47 (+0.48)	96.83 (-0.29)	97.60 (-0.07)	96.79 (-1.23)	97.57 (-0.78)	96.83 (-1.61)	97.59 (-1.13)	96.83 (-2.25)	97.70 (-1.58)
NN	91.82 (-3.27)	92.85 (-3.35)	96.28 (-1.25)	96.55 (-1.44)	97.00 (-0.12)	97.29 (-0.38)	97.61 (-0.41)	97.81 (-0.54)	98.04 (-0.4)	98.21 (-0.51)	98.25 (-0.64)	98.44 (-0.84)
CNN-2	90.49 (-4.6)	91.16 (-5.04)	95.74 (-1.79)	96.11 (-1.88)	93.98 (-3.14)	94.89 (-2.78)	96.45 (-1.57)	96.73 (-1.62)	96.78 (-1.66)	97.27 (-1.45)	97.63 (-1.45)	97.83 (-1.45)
CNN-1	95.09	96.20	97.53	97.99	97.12	97.67	98.02	98.35	98.44	98.72	99.08	99.28

10-fold cross-validation of static SSVEP classification, changing the amount of training data with 1,500 test data. Low indicates subjects who have a low CCA accuracy (under 80% in the ambulatory SSVEP, i.e., subjects S3–7). Parentheses indicate the differences in accuracy when compared with CNN-1.

**Fig 8:**
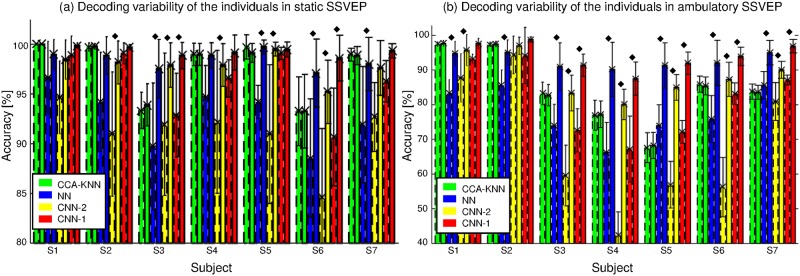
Decoding variability across individuals. Decoding variability of individuals at the minimum and maximum number of data samples (dash and solid line, respectively) in static (a) and ambulatory (b) SSVEP environments.

Fig 8(a) shows the decoding variability of the individual subjects at the minimum (dash) and maximum (solid line) number of data samples; here, a diamond indicates a 5% or more increase in classification rates. Clearly, all subjects achieved increased accuracies with neural network models. Specifically, low-performing subjects (S3–7) show a higher increase than other subjects (S1 and S2). Subjects S2 and S3 only increase in the CCA-KNN method.

### Ambulatory SSVEP

Table 3 considers the 10-fold cross-validation results for the ambulatory SSVEP setup when the number of training data is 67,500, with 7,500 test data for all subjects. CNN-1 showed the best classification accuracy of all subjects in each classifier. Even the low-performing subjects showed the highest accuracy with CNN-1. The competing classifier models showed a more pronounced performance deterioration owing to the higher artifact presence in the ambulatory environment (see Figs 5 and 6 in [14]).

**Table 3:** 10-fold cross-validation results of individual subjects at the maximum training data (i.e., 67,500) for ambulatory SSVEP classification.

	S1	S2	S3	S4	S5	S6	S7	Low	All
CCA	92.43 (-5.24)	90.40 (-8.41)	73.10 (-18.3)	65.46 (-22.05)	43.99 (-47.99)	69.90 (-23.48)	75.05 (-21.85)	65.50±12.56 (-26.85)	72.90±16.29 (-21.13)
MSI	94.02 (-3.65)	92.35 (-6.46)	78.31 (-13.09)	69.22 (-18.29)	43.67 (-48.30)	71.00 (-22.95)	77.80 (-19.11)	68.00±14.18 (-24.35)	75.19±16.89 (-18.84)
CCA-KNN	97.69±0.82 (+0.02)	97.53±0.78 (-1.28)	82.77±3.05 (-8.63)	77.27±3.82 (-10.24)	68.25±3.76 (-23.73)	85.43±2.57 (-7.95)	83.58±2.05 (-13.32)	79.46±6.96 (-12.87)	84.65±10.52 (-9.38)
NN	94.77±3.48 (-2.9)	95.11±3.58 (-3.7)	91.03±6.82 (-0.37)	90.20±7.82 (+2.69)	91.35±6.51 (-0.63)	92.07±6.54 (-1.31)	95.03±3.54 (-1.87)	91.94±1.86 (-0.41)	92.80±2.11 (-1.23)
CNN-2	95.76±4.47 (-1.91)	96.99±2.51 (-1.82)	83.36±5.16 (-8.04)	80.09±4.4 (-7.42)	84.96±3.71 (-7.02)	87.32±4.92 (-6.06)	90.07±2.42 (-6.83)	85.16±3.80 (-7.19)	88.36±6.30 (-5.67)
CNN-1	97.67±1.50	98.81±0.74	91.40±3.21	87.51±4.8	91.98±3.23	93.38±2.65	96.90±1.92	92.35±3.46	94.03±4.04

10-fold cross-validation results of ambulatory SSVEP classification for 67,500 training data and 7,500 test data. Low indicates subjects who have a low CCA accuracy (under 80%, i.e., subjects S3–7). In parentheses, the accuracy differences compared with CNN-1 are indicated.

Table 4 and Fig 7 (bottom) presents the 10-fold cross-validation results for the ambulatory SSVEP classification as a function of a changing number of training data with 7,500 test data. In particular, Fig 7 (bottom) shows the averaged accuracy of each classifier with increasing training data for all subjects (c) and low-performance subjects (d). One asterisk indicates the 5% significance level (compared to 67,500 training data samples), whereas two asterisks denote the 1% significance level. As expected, analysis confirms the performance gains of the neural networks as training data increases, even if the data contain large artifacts. Fig 8(b) confirms the findings of the static setting for the more artifact-prone ambulatory setting.

**Table 4:** 10-fold cross-validation of ambulatory SSVEP classification by the quantity of training data.

	11,250		22,500		33,750		45,000		56,250		67,500	
	Low	All	Low	All	Low	All	Low	All	Low	All	Low	All
CCA-KNN	79.51 (+3.04)	84.64 (+3.24)	79.28 (-3.11)	84.49 (-1.69)	79.16 (-6.43)	84.41 (-4.38)	79.29 (-8.56)	84.52 (-6.14)	79.36 (-12.09)	84.58 (-8.72)	79.46 (-12.87)	84.65 (-9.38)
NN	75.15 (-0.88)	77.81 (-3.59)	82.63 (+0.24)	84.61 (-1.57)	86.39 (-0.8)	87.93 (-0.86)	89.21 (+1.36)	90.35 (-0.31)	91.04 (-0.41)	91.96 (-1.34)	91.94 (-0.41)	92.80 (-1.23)
CNN-2	59.21 (-17.26)	68.31 (-13.09)	73.77 (-8.62)	79.78 (-3.4)	78.95 (-6.64)	83.99 (-4.8)	82.52 (-5.33)	85.77 (-4.89)	83.91 (-7.54)	87.75 (-5.55)	85.16 (-7.19)	88.36 (-5.67)
CNN-1	76.47	81.40	82.39	86.18	85.59	88.79	87.85	90.66	91.45	93.30	92.35	94.03

10-fold cross-validation of the ambulatory SSVEP classification when changing the number of training data with 7,500 test data. Low indicates subjects who have a low CCA accuracy (under 80%, i.e., subjects S3–7). In parentheses, the accuracy differences of the methods compared with CNN-1 are indicated.

Compared with the static SSVEP in CNN-1, larger training data samples are required for the ambulatory SSVEP to accomplish high accuracy (classification performance of more than 90% at 56,250 training data samples). For the static SSVEP setup, 96.20% accuracy could be achieved using only 2,250 training data and 99.28% accuracy was achieved using 13,500 samples. In contrast, 81.40% accuracy and 94.03% accuracy were achieved in the ambulatory condition when 11,250 and 67,500 training data were used, respectively.

**Fig 9:**
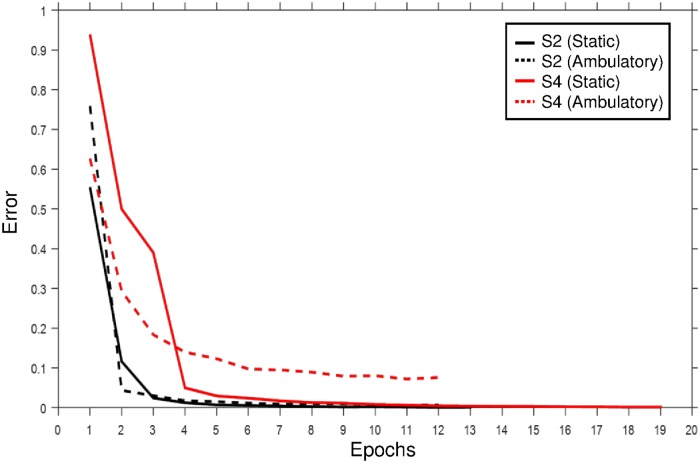
Learning curve for subjects S2 and S4. Learning curve of subjects S2 (black) and S4 (red) using static (solid) and ambulatory (dash line) SSVEP environments in CNN-1.

Fig 9 shows the learning curves of subjects S2 (black) and S4 (red) in static (solid) and ambulatory (dash line) SSVEP environments in CNN-1. The learning iteration of subject S2 stops at the 13th and 12th epochs, whereas the iteration of subject S4 stops at the 19th and 12th epochs in the datasets (subject S2 records the best performance with CNN-1 and subject S4 has the lowest performance in the ambulatory SSVEP.)

The appearance of the kernels differs for each individual because the network training is subject-dependent. Unfortunately, there is no obvious and simple interpretation linked to physiology or the experimental task (see S3 Fig which describes the convolutional kernels learned from CNN-2 using ambulatory SSVEP data for subject S2 (top), and subject S3 (bottom)).

**Fig 10:**
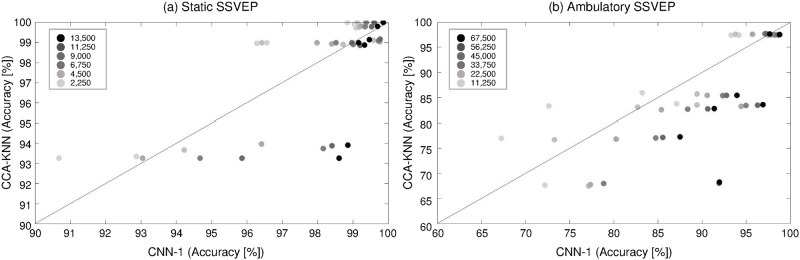
Decoding trends of CNN-1 compared with CCA-KNN for the individual subjects. Decoding trends of CNN1 compared with CCA-KNN by the number of training data in static SSVEP (a) and ambulatory (b) SSVEP environments. The circles indicate a higher quantity of training data with darker color. The more training data were given, the better CNN-1 performed (more black circles are located on the right side).

Fig 10 shows the decoding trends of CNN-1 compared with CCA-KNN for individuals as a function of the number of training data in the static (a) and ambulatory (b) SSVEP setups. The darker circles indicate more training data. As more training data were given, we observed that the performance of the CNN-1 increased consistently and was more pronounced under the ambulatory condition (more black circles are located on the right side). However, individual CCA-KNN decoding accuracies stay relatively stable, meaning that the accuracy of the CCA-KNN is almost independent of the amount of training data in our experimental conditions.

### Feature representation

**Fig 11:**
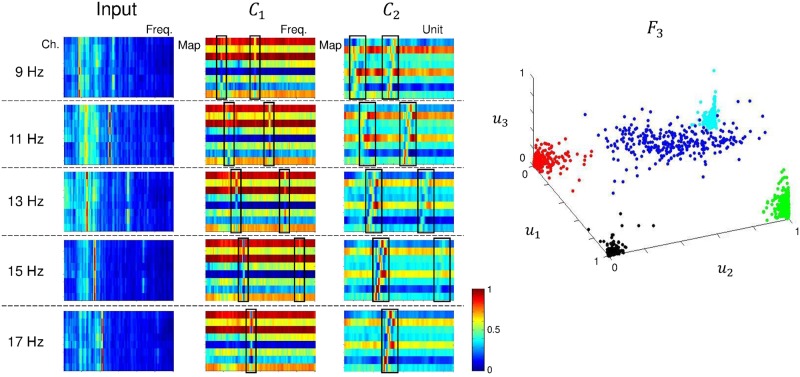
Feature representation of CNN-2 using static SSVEPs for subject S1. Representation of the average features of each layer in CNN-2 using static SSVEP data. In layer *F*_3_, blue is 9 Hz; red, 11 Hz; green, 13 Hz; black, 15 Hz; and cyan, 17 Hz.

**Fig 12:**
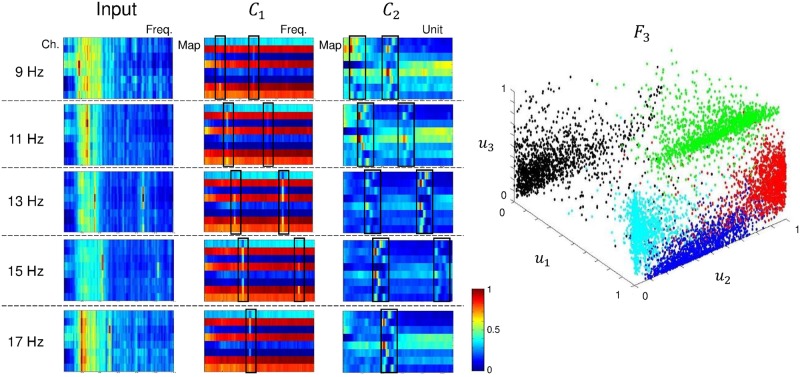
Feature representation of CNN-2 using ambulatory SSVEP for subject S1. Representation of the average features of each layer in CNN-2 using ambulatory SSVEP data. In layer *F*_3_, blue is 9 Hz; red, 11 Hz; green, 13 Hz; black, 15 Hz; and cyan, 17 Hz.

Analyzing and understanding classification decisions in neural networks is valuable in many applications, as it allows the user to verify the system’s reasoning and provides additional information [54, 62]. Although deep learning methods are very successfully solving various pattern recognition problems, in most cases, they act as a black box, not providing any information about why a particular decision was made. Hence, we present the feature representation from the CNN-2 architecture. The averaged features of each layer using static and ambulatory SSVEPs are shown in Figs 11 and 12, respectively. In both cases, the networks focus on the stimulus frequency components. For learning in layer *C*_1_, we used a 1 × *N*_*ch*_ convolutional kernel, which can give channel-wise (spatial) weight. The *C*_2_ layer used an 11 × 1 convolutional kernel to detect frequency (spectral) information. The frequency components that were most discriminated by the convolutional layers were highlighted using black-lined boxes. With the exception of the 17 Hz class, the corresponding stimulus frequencies were enforced through iterative training. We conjecture that the absence of second harmonics (34 Hz) for the 17 Hz SSVEPs results from low magnitude when compared with lower frequencies or outside the boundary of the ranges in the *C*_2_ layer. In the second convolutional layer, the patterns were spread out (and slightly smoothed) when compared to the first convolutional layer. The *F*_3_ layer is composed of three units that we plotted with each unit as an axis direction. The 3D plot shows that all classes are distinguished nicely. Therefore, we can conclude that the CNN architecture is able to appropriately extract the meaningful frequency information of SSVEP signals. To compare the feature distributions with CCA-KNN, we show a scatter plot using CCA-KNN in S4 Fig. The features were extracted with CCA and classified using KNN when *k* = 3 for subject S6 (85%). Test data were plotted on *ρ*_*f*_1__, *ρ*_*f*_2__ and *ρ*_*f*_3__ axes. Blue, red, green, black, and cyan circles indicate 9 Hz, 11 Hz, 13 Hz, 15 Hz, and 17 Hz, respectively. Note that the feature dimension is actually 5 (the number of classes), therefore we only used the *ρ*_*f*_1__, *ρ*_*f*_2__ and *ρ*_*f*_3__ projection to visualize feature distributions in the plot. However, the classes are clearly not as well spread apart when compared with CNN-2.

## Conclusion

BMI systems have shown great promise, though significant effort is still required to bring neuroprosthetic devices from the laboratory into the real world. In particular, further advancement in the robustness of brain signal processing techniques is needed [63, 64]. In this context, constructing reliable BMI-based exoskeletons is a difficult challenge owing to the various complex artifacts spoiling the EEG signal. These artifacts may be induced differently depending on subject population and may in particular be caused by suboptimal EEG measurements or broadband distortions due to movement of the exoskeleton. For example, while walking in the exoskeleton a subject’s head may move, which can give rise to swinging movements in the line between the electrodes and EEG amplifiers, leading to disconnections or high impedance measurements. Furthermore, significant challenges still exist in the development of a lower-limb exoskeleton that can integrate with the user’s neuromusculoskeletal system. Although these limitations exist, a brain-controlled exoskeleton may eventually be helpful for end-user groups.

The current study made a step forward toward more robust SSVEP-BMI classification. Despite the challenges imposed on signal processing by a lower-limb exoskeleton in an ambulatory setting, our proposed CNN exhibited promising and highly robust decoding performance for SSVEP signals. The neural network model was successfully evaluated offline against standard SSVEP classification methods on SSVEP datasets from static and ambulatory tasks. The three neural networks (CNN-1, CNN-2, and NN) showed increased performance in both environments when sufficient training data were provided. CNN-1 outperformed all other methods; the best accuracies achieved by CNN-1 were 99.28% and 94.03% in static and ambulatory conditions, respectively. Other methods (CCA-KNN, NN, CNN-2) showed high accuracy in the static environment, but only CNN-1 recorded smallest low performance deterioration for the ambulatory SSVEP task. CNN-1’s complexity is low because it has a comparatively simple structure (few layers, maps, and units) and the weights in the convolution layers are shared for every unit within one map, effectively reducing the number of free parameters in the network. Our application is far from being data rich (N ≤ 67,500); therefore, we adopted neither pre-trained model, dropout, nor pooling methods, yet our relatively simple architecture worked efficiently after a brief training period. Overall, the proposed method has advantages for real-time usage and it is highly accurate in the ambulatory conditions. Furthermore, our method can increase in accuracy with more data, if available. Note that we consider subject-dependent classifiers for decoding, which reflects the fact that individuals possess highly different patterns in their brain signals. From the kernel analysis, we therefore found—as expected—that the convolutional kernels were different for each individual. We also demonstrate the feature representations, as implemented using a bottleneck layer in CNN-2. The CNN classifiers could determine the most discriminative frequency information for classification, nicely matching the stimulus frequencies of the respective SSVEP classes.

So far, our study has only successfully tested the performance of CNN classifiers for offline data. Future work will also develop a real-time CNN system that can control a lower-limb exoskeleton based on the proposed method and evaluate its performance with healthy volunteers as well as for end-user groups to investigate their use in gait rehabilitation. We will investigate subject-independent classification using CNNs. A subject-independent CNN-based classifier may be more efficient system because it could reduce long training times.

## Supporting information

S1 Fig Examples of input data. Randomly selected input data and averaged data of (a) static and (b) ambulatory SSVEPs for a representative subject S7. Red boxes indicate the frequency location corresponding to stimulus frequencies.
(PDF)

S2 Fig Accuracy differences in 10-fold cross-validation performance using static (top) and ambulatory (bottom) SSVEPs as the number of training data increases. (a) Accuracy differences for all subjects in static SSVEP. (b) Accuracy differences for low-performance subjects in static SSVEP. (c) Accuracy differences for all subjects in ambulatory SSVEP. (d) Accuracy differences for low-performance subjects in ambulatory SSVEP.
(PDF)

S3 Fig Kernel appearance. Kernels of layer *C*_1_ (left) and *C*_2_ (right) in CNN-2 using ambulatory SSVEPs for S2 (top) and S3 (bottom).
(PDF)

S4 Fig A feature representation of CCA-KNN. Features were extracted from CCA and classified using KNN with *k* = 3 for subject S6. Test data were plotted along the *ρ*_*f*_1__, *ρ*_*f*_2__ and *ρ*_*f*_3__ axes. Blue, red, green), black, and cyan are 9, 11, 13, 15, and 17 Hz, respectively.
(PDF)
