# Supplementary figures and images for: A convolutional neural network for steady state visual evoked potential classification under ambulatory environment
Source: PLoS One. 2017 Feb 22;12(2):e0172578. doi: 10.1371/journal.pone.0172578 (PMC5321422; doi:10.1371/journal.pone.0172578)

(a) Input data samples of static SSVEPs

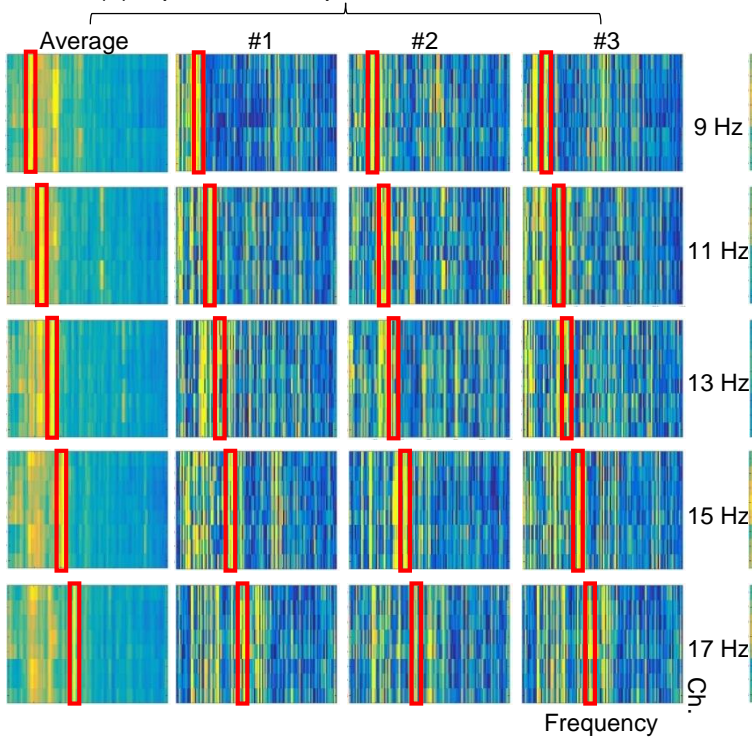

(b) Input data samples of ambulatory SSVEPs

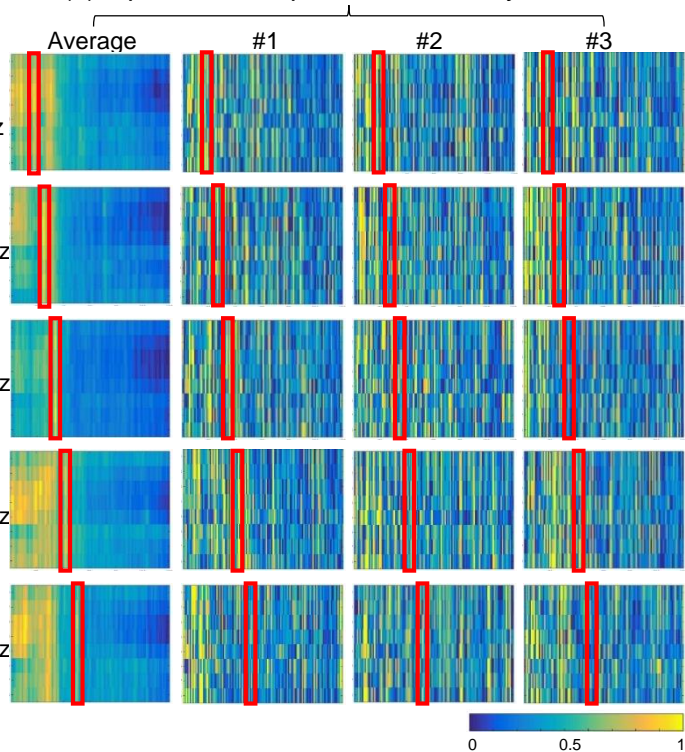

Supplement: S1 Fig — Randomly selected input data and averaged data of (a) static and (b) ambulatory SSVEPs for a representative subject S7. Red boxes indicate the frequency location corresponding to stimulus frequencies. (PDF) [file pone.0172578.s001.pdf]

(a) All subjects

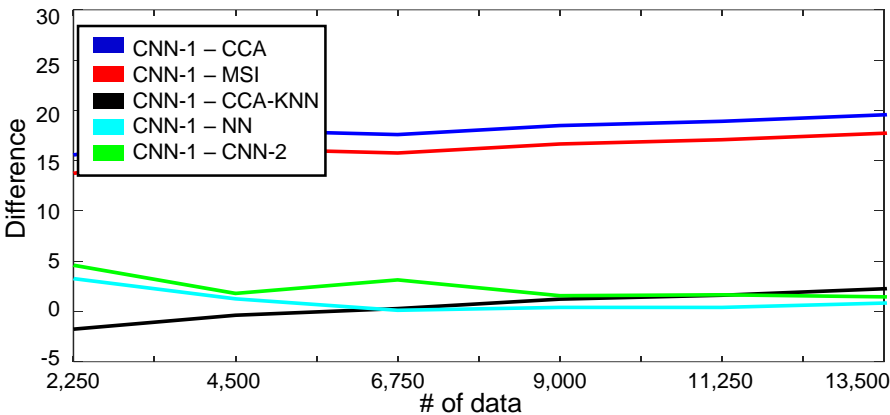

(b) Low-performing subject

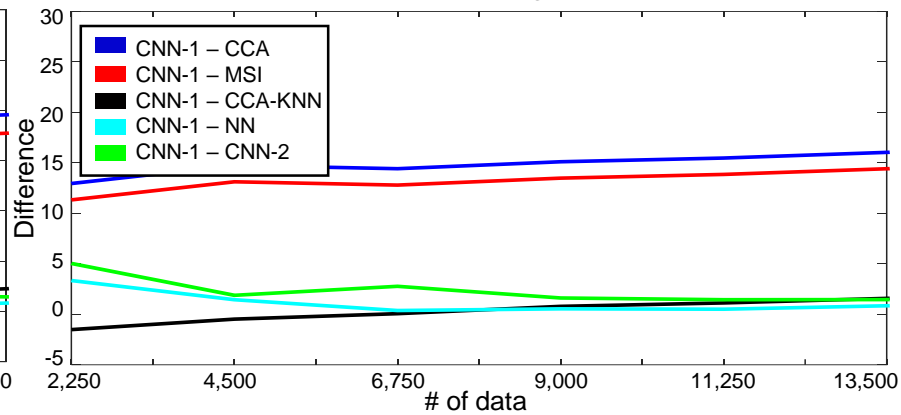

(c) All subjects

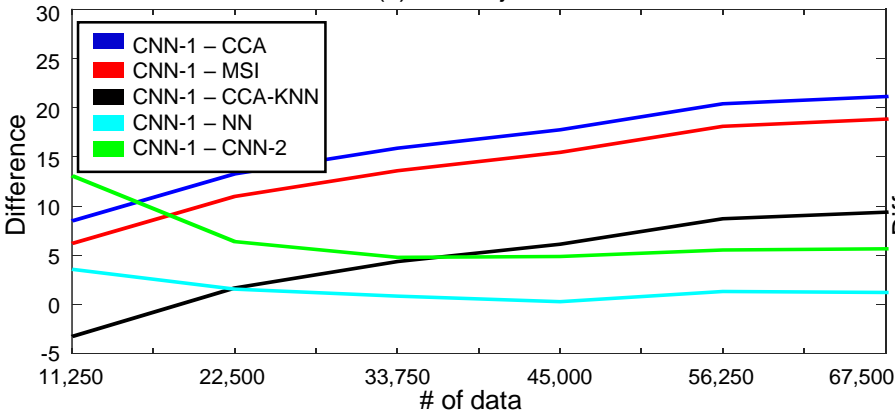

(d) Low-performing subject

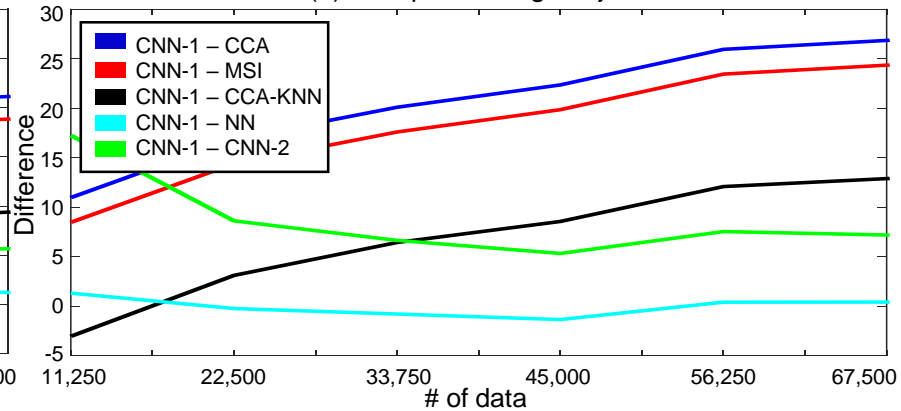

Supplement: S2 Fig — (a) Accuracy differences for all subjects in static SSVEP. (b) Accuracy differences for low-performance subjects in static SSVEP. (c) Accuracy differences for all subjects in ambulatory SSVEP. (d) Accuracy differences for low-performance subjects in ambulatory SSVEP. (PDF) [file pone.0172578.s002.pdf]

Kernels obtained with CNN-2 in  $C_1$  (left) and  $C_2$  (right) layers for subject S2 (top) and S3 (bottom)

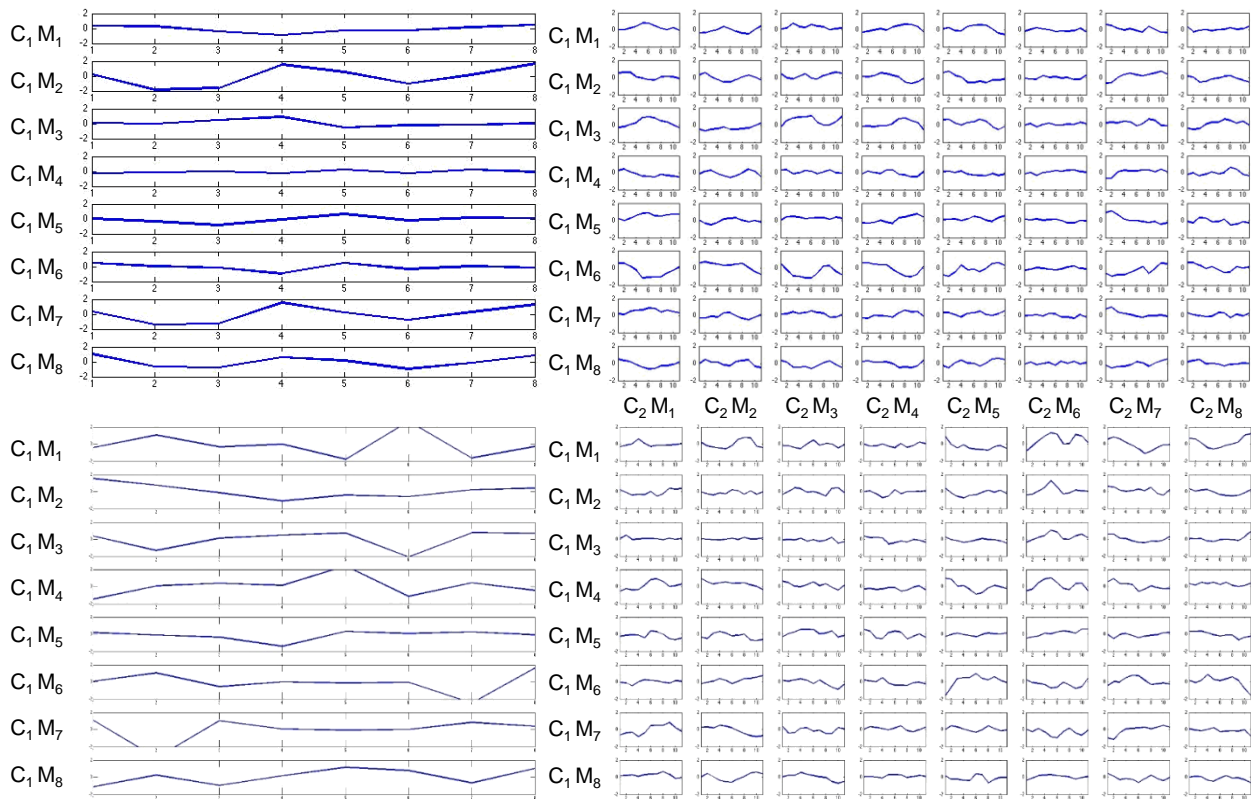

Supplement: S3 Fig — Kernels of layer C1 (left) and C2 (right) in CNN-2 using ambulatory SSVEPs for S2 (top) and S3 (bottom). (PDF) [file pone.0172578.s003.pdf]

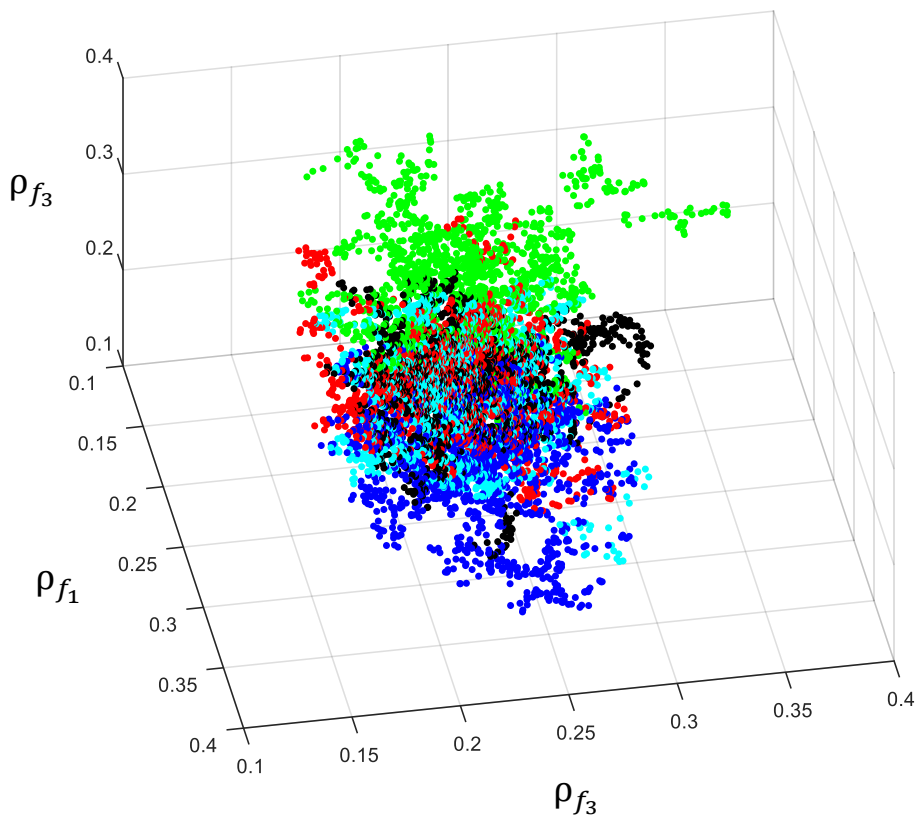

Supplement: S4 Fig — Features were extracted from CCA and classified using KNN with k = 3 for subject S6. Test data were plotted along the ρf1, ρf2 and ρf3 axes. Blue, red, green), black, and cyan are 9, 11, 13, 15, and 17 Hz, respectively. (PDF) [file pone.0172578.s004.pdf]
